# Developing integrated care pathways for atopic dermatitis—Challenges and unmet needs

**DOI:** 10.1002/clt2.12236

**Published:** 2023-03-26

**Authors:** Torsten Zuberbier, Lisa A. Beck, Anna Bedbrook, Marjolein de Bruin‐Weller, Jean Bousquet, Michael Cork, Nikolaos Douladiris, Norito Katoh, Charlotte G. Mortz, Thomas Werfel, Francuzik Wojciech, Andreas Wollenberg, Kristina Siemens, Katarina Stevanovic, Margitta Worm, AD‐ICPs Working Group

**Affiliations:** ^1^ Institute of Allergology Charité‐Universitätsmedizin Berlin Berlin Germany; ^2^ Fraunhofer Institute for Translational Medicine and Pharmacology ITMP Allergology and Immunology Berlin Germany; ^3^ University of Rochester Medical Center Rochester New York USA; ^4^ ARIA Montpellier France; ^5^ MASK‐air Montpellier France; ^6^ Department of Dermatology and Allergology National Expertise Center for Atopic Dermatitis University Medical Center Utrecht Utrecht The Netherlands; ^7^ University Hospital of Montpellier Montpellier France; ^8^ Sheffield Dermatology Research IICD University of Sheffield Sheffield UK; ^9^ Allergy Department 2nd Paediatric Clinic National & Kapodistrian University of Athens Athens Greece; ^10^ Department of Dermatology Kyoto Prefectural University of Medicine Kyoto Japan; ^11^ Department of Dermatology and Allergy Centre Odense Research Centre for Anaphylaxis (ORCA) Odense University Hospital University of Southern Denmark Odense C Denmark; ^12^ Division of Immunodermatology and Allergy Research Department of Dermatology and Allergy Hannover Medical School Hannover Germany; ^13^ Division of Allergy and Immunology Department of Dermatology, Venerology and Allergy Charité‐Universitätsmedizin Berlin Berlin Germany; ^14^ Department of Dermatology and Allergy Ludwig‐Maximilian‐University Munich Germany; ^15^ Department of Dermatology Free University Brussels University Hospital Brussels Brussels Belgium; ^16^ Department of Women and Children's Health Faculty of Life Sciences and Medicine King's College London London UK

**Keywords:** atopic dermatitis, eczema, guidance, integrated care pathways (ICPs), multidisciplinary, prevention, treatment

## Abstract

**Background:**

GA^2^LEN‐ADCARE is a branch of the largest multidisciplinary network of research centres and clinical care in allergy and asthma, GA^2^LEN, focussing on the field of atopic dermatitis (AD). AD is a chronic inflammatory skin disease with high burden and many comorbidities requiring different levels of treatment. The need for aligned information from all involved healthcare providers led to the discussion of an integrated care pathway (ICP) plan for AD patient care involving all stakeholders and considering the complexity and variability of the disease, with a particular focus placed on the large number of patients with milder forms of AD.

**Methods:**

The GA^2^LEN ADCARE network and all stakeholders, abbreviated the AD‐ICPs working group, were involved in the discussion and preparation of the AD‐ICPs during a series of subgroup workshops and meetings in years 2020 and 2021.

**Results:**

Here we discuss the unmet needs in AD, the methodology for devising an AD‐ICP and the ICP action plan.

**Conclusion:**

The GA^2^LEN ADCARE network has outlined the unmet needs in AD and provided an action plan for devising AD‐ICPs, considering the complexity and variability of the disease.

## 1 INTRODUCTION

The Global Allergy and Asthma European Network (GA^2^LEN), originally started in 2004 as the European Union network of excellence in collaboration with European Academy of Allergy and Clinical Immunology (EAACI). It is the largest multidisciplinary network of research centres and clinical care in allergy and asthma. Within GA^2^LEN, there are subnetworks for different areas of allergy. These subnetworks collaborate in research and educational activities, as well as in the exchange of experience on novel and emerging approaches for the treatment of severely affected patients. One of the subnetworks is called ADCARE and is for atopic dermatitis (AD).

AD is a chronic inflammatory skin disease representing a lifelong disposition with variable clinical manifestations and expressivity. Together with asthma and allergic rhinitis, AD is among the most common chronic diseases and is the leading non‐fatal health burden attributable to skin diseases. AD inflicts a substantial psychosocial burden on patients and their relatives and increases the risk of comorbidities such as food allergy, asthma, allergic rhinitis, other immune‐mediated inflammatory diseases and mental health disorders.^1^, ^2^, ^3^

Here we describe the unmet need and the methodology of an integrated care pathway (ICP) document for AD, structuring multidisciplinary plans for patient care, integrating recommendations from evidence‐based guidelines and facilitating their application to clinical practice.

## 2 CHALLENGES AND UNMET NEEDS IN THE MANAGEMENT OF AD AS A RATIONAL FOR ICPs

The management of AD is challenged by the complexity of the disease, involving multiple genetic and environmental factors interlinked through immunoglobulin E (IgE)‐associated and non‐IgE‐associated mechanisms. The spectrum of clinical patterns and severity levels is wide with a high age‐dependent and interindividual variability.^1^, ^4^ Hence, personalised, predictive, preventative and participatory (P4) approaches have been advocated.^5^ Precision medicine is of broad relevance for the management of AD in the context of a better selection of treatment responders, risk prediction and design of disease‐modifying strategies.^6^

A comprehensive guideline for the treatment of adults and children with AD was developed as a joint interdisciplinary European project, including physicians from all relevant disciplines as well as patients. It is a consensus‐based S2k‐guideline and was last updated in 2018.^4^, ^7^ This guideline was upgraded to the S3 level and was published in 2022 (S2k and S3 are different stages of a guideline development according to the German Instrument for Methodological Guideline Appraisal. S3 is a highest level of evidence, consensus‐based guideline distinction. More information can be found on www.awmf.org).^8^, ^9^ Globally, the management of AD is challenged by poor adherence to treatment and substantial differences in the diagnostic and therapeutic strategies practiced in different countries. Recognising these, the EAACI and the American Academy of Allergy, Asthma and Immunology (AAAAI) developed the practical allergy (PRACTALL) initiative aiming to harmonise the European and American approaches to best allergy practice and science.^6^ Moreover, the European Task Force on Atopic Dermatitis (ETFAD) produces a consensus statement of AD management once every 5 years^10^

While guidelines and consensus reports generally provide valuable guidance focussing on the more severe spectrum of AD, patients with milder disease are less considered despite representing a group large in numbers.^4^, ^9^

**FIGURE 1:**
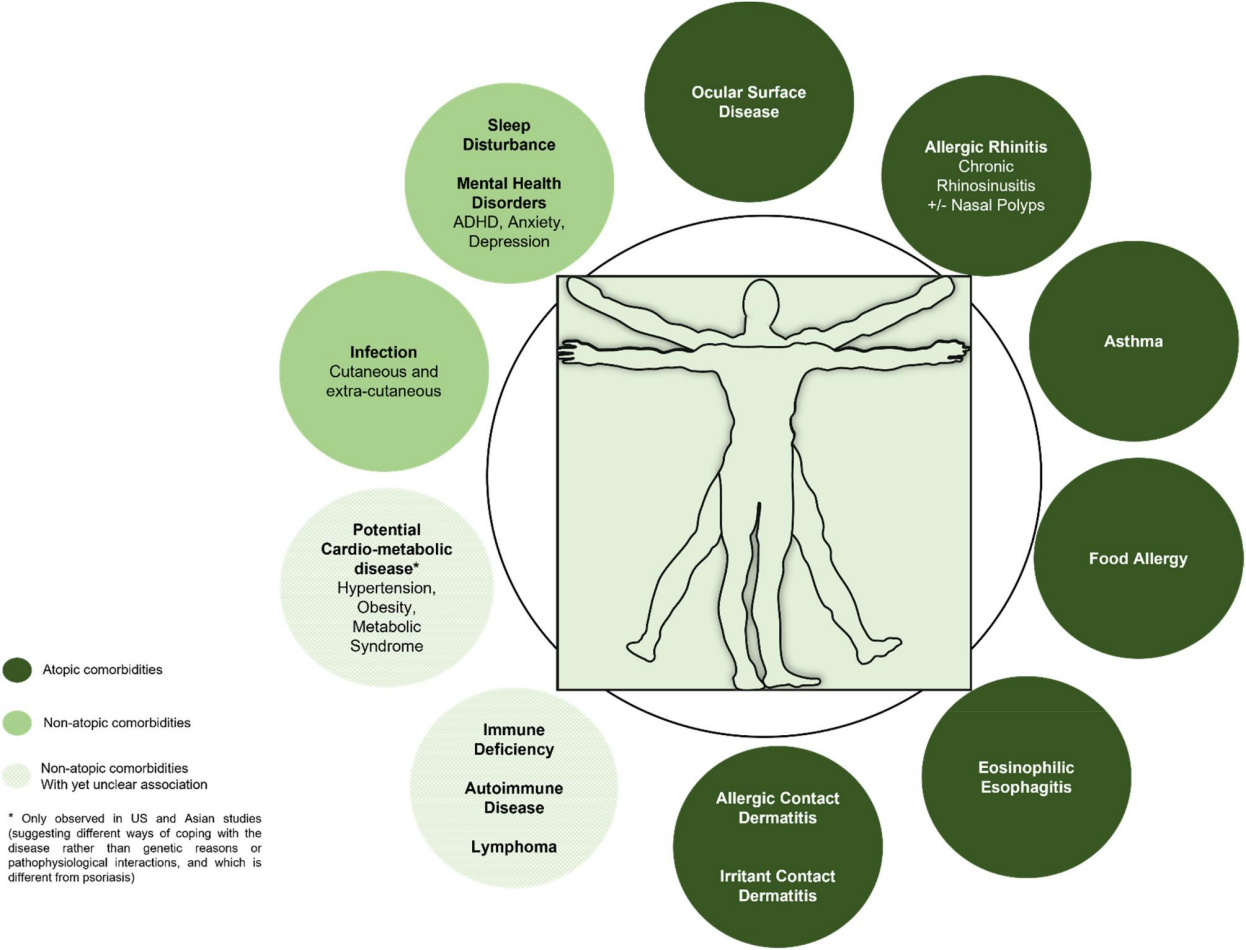
More than a skin disease—atopic and non‐atopic comorbidities in AD.

AD and other allergic diseases such as asthma and allergic rhinitis tend to cluster and patients present concomitant or consecutive diseases—such as ocular morbidity in the form of conjunctivitis and keratitis—which are more frequently compared with the general population.^11^ Also, numerous non‐allergic comorbidities have been associated with AD, suggesting that it may be more of a systemic disorder than previously recognised (Figure 1). Among these are extra‐cutaneous infections, neuropsychiatric conditions, obesity (observed in Asia and the US but not in Europe and Canada), metabolic syndrome (observed in Asia and the US but not in Europe and Canada), autoimmune disease and lymphoma (the latter being however controversially discussed).^12^ Patients with multimorbidity have complex health needs but, due to traditional disease‐oriented approaches, they often face a fragmented form of care.^13^ Pregnant and breastfeeding women with AD are another group of patients needing special attention regarding choice of treatment.^14^ Managing physicians should therefore consider potential allergic and non‐allergic comorbidities and conditions in a holistic approach.

AD patients are treated on many different levels comprising self‐management and support from pharmacists, general practitioner (GPs), specialists and other healthcare providers. A large proportion of all patients with AD suffer from a mild form of the disease and could be managed mainly by the GP and nurse practitioners. Pharmacists are a further valuable source of support, especially for patients with mild disease, and should therefore be included in AD care to a greater extent. A recent ETFAD position statement summarises the quality of care for AD management as a function of expert level and resources ranging from GPs to specialists.^15^ In this context, the need for aligned information of all involved healthcare providers becomes important.

Poor adherence to treatment is a major factor limiting treatment outcomes in patients with AD. Education plays a major role with the goal of an empowered patient or caregiver respectively. This empowerment should enable the identification of individual symptoms, trigger factors and the need for treatment but also the need to seek professional care in adequate time.^16^, ^17^ Structured education programmes have been efficacious in terms of numerous outcomes—for both paediatric and adult AD patients—in controlled studies.^18^, ^19^ In this context, several studies also suggest that community pharmacist‐led interventions contribute to improved medication adherence and better disease control.^20^

On a larger scale, the burden and cost of allergic and chronic respiratory diseases are increasing rapidly in all societies, with the challenge to deliver modern health care effectively. From this arises a need to support the transformation of the healthcare system into integrated care with organisational health literacy.^21^, ^22^

Hence, in addition to guidelines and consensus reports, ICPs for all involved stakeholders are warranted in consideration of the complexity and variability of the disease and with a particular focus on the large number of patients with milder forms of AD. A multidisciplinary approach including early attention to atopic and psychiatric comorbidities is needed to overcome the painful, itching and stigmatising flare‐ups as well as their impact on quality of life, aiming for a fast and effective management of exacerbations as well as long‐term disease control.^4^, ^16^, ^17^, ^23^, ^24^

## 3 INTEGRATED CARE PATHWAYS IN ATOPIC DISEASES

ICPs are structured multidisciplinary plans for patient care integrating recommendations from evidence‐based guidelines and facilitating their application to clinical practice. They have the potential to enhance guideline recommendations by combining interventions, integrating quality assurance and describing coordination of care.^25^

AIRWAYS ICPs is an example of a multi‐disciplinary approach to reduce the burden of chronic respiratory diseases, their mortality and multimorbidity and, in the long term, to promote active and healthy ageing (AHA).^26^, ^27^ Similarly to AD, allergic rhinitis and asthma multimorbidity represents a major world‐wide chronic disease burden complicated by a general poor adherence to treatment. Hence, Allergic Rhinitis and its Impact on Asthma (ARIA) has promoted the integration of its recommendations in ICPs using mobile technology to reinforce self‐management and the implementation of guidelines.^28^, ^29^, ^30^ This approach is supported by results from a meta‐analysis indicating clinical effectiveness of mobile apps in improving asthma control.^31^

The ARIA digitally‐enabled, integrated, person‐centred care for rhinitis and asthma multimorbidity using real‐world‐evidence could guide the way to similar pathways for the field of AD. Embedding these together with pathways for further chronic diseases in one unifying digital application—provided on a government level, in different languages, on a cloud server and with full global accessibility—is an unmet need on its way to harmonising the management of nomadic working citizens.

## 4 DEVELOPING ICPs FOR ATOPIC DERMATITIS

GA^2^LEN ADCARE has taken the lead, informing EAACI, European Academy of Dermatology and Venereology (EADV) and World Allergy Organization (WAO) upfront about the initiative, requesting their involvement in the future and offering to send delegates. Other societies will be informed and asked for involvement at a later stage. The core of the AD‐ICPs working group consists of the speakers of an online conference held on 26 March 2020. This conference was originally planned as a physical meeting. Due to the COVID‐19 pandemic, the meeting was held as an online conference initiating the project. A further online meeting was held on 15 June 2020, followed by a hybrid meeting on 13 August 2021.

### 4.1 Objectives

The general objective of AD‐ICPs is to develop pragmatic and practical support to tackle the disease and its comorbidities globally.

(1) AD‐ICPs will not duplicate existing professional guidelines or European Union (EU)/national prevention programmes but will strengthen them where appropriate. They will aim to help improve the adherence to guideline recommendations by adjusting them in a dynamic way to different real‐world conditions including availability of medical services, drugs and reimbursement of drugs.
(2) As the hallmark of ICPs is the translation of guidelines into clinical practice, the AD‐ICPs are meant to be concise and easy to understand for readers who are not specialists in the field.
(3) A holistic approach will be strived for (i) to improve multidisciplinary communication, including primary care and pharmacists, (ii) to improve clinician–patient communication and patient satisfaction and (iii) to empower patients and their caregivers. Accordingly, AD‐ICPs will be designed to be carried out by a multidisciplinary team including physicians, pharmacists, specialised nurses and allied healthcare professionals.
(4) AD‐ICPs will consider technology‐assisted patient activation by mobile health tools to enhance self‐management, adherence to guidelines and shared decision making among the specialists.
(5) The aim of AD‐ICPs is (i) to unify themes and an overall potential to gain political leverage in the current setting when new therapies emerge and (ii) to better understand and manage the spectrum of care for patients with AD in international countries and regions.

Specific objectives recorded in the forefront of developing the ICPs are summarised in Table 1.

**TABLE 1:** Specific objectives recorded in the forefront of developing ICPs for AD.

1	To compile a brief and concise tool that is easy to understand for people with and without medical background
2	To give an overview of the existing guidelines, consensus statements and ICPs as well as real‐world data
3	To focus on the present situation and evidence but also to include visions for the future
4	To strengthen prevention and health promotion for AD
5	To mainly cover diagnostic and treatment of patients with AD, aiming to discuss detailed guidance on prevention and health promotion separately
6	To aid the diagnosis of AD
7	To provide a structured approach to treatment strategies including OTC therapies, with a focus on the interventions that are mainly used
8	To consider all AD comorbidities as significant, such as psychiatric morbidity. This is seen not only in severe AD but also in moderate AD. As an example, ocular morbidity can exist in patients with only eyelid dermatitis (very low EASI)
9	To have a particular focus on mild to moderate AD, as severe AD is already covered in detail in AD guidelines. The ICPs will refer to these when appropriate
10	To stratify patients with severe AD
11	To understand AD and the different comorbidities in subgroups such as children, adolescents and older people, and to develop relevant criteria to guide their management
12	To understand and overcome barriers in a holistic patient‐centred approach managing AD, including somatic, psychosomatic and psychiatric comorbidities such as anxiety and depression as well as the impact of the disease on work
13	To investigate and consider different practices in different countries, for example, the role of pharmacists in patient care
14	To consider all parties involved in the patients' care, including the role of para‐medical staff such as physician assistants, dermatological nurses, nurse specialists, social workers and assistants in the GP's office, considering their roles, limitations and their place within the multidisciplinary team
15	To specifically develop a unique educational module for pharmacists on AD recognition, the use of emollients, specific treatments and disease monitoring. As a first step, the role of the pharmacist in patient management needs to be identified in different countries
16	To develop ICPs for rhinitis, asthma and ocular comorbidity across the life cycle in AD, inter linking with the existing AIRWAYS ICPs
17	To determine whether mobile health tools like MASK‐air for rhinitis and asthma comorbidity could be applied, redesigning care pathways also for AD patients
18	To investigate and discuss unmet needs such as the cultural and social aspects of the disease starting in childcare and school but also in nursing homes, in a project centred on the patient
19	To implement multi‐sectoral, multi‐country initiatives involving the ADCARE network, creating solutions for trials and registries to investigate real‐life settings

Abbreviations: EASI, Eczema Area and Severity Index; GP, general practitioner; MASK, Mobile Airways Sentinel Network; OTC, over the counter.

### 4.2 Stakeholders

**FIGURE 2:**
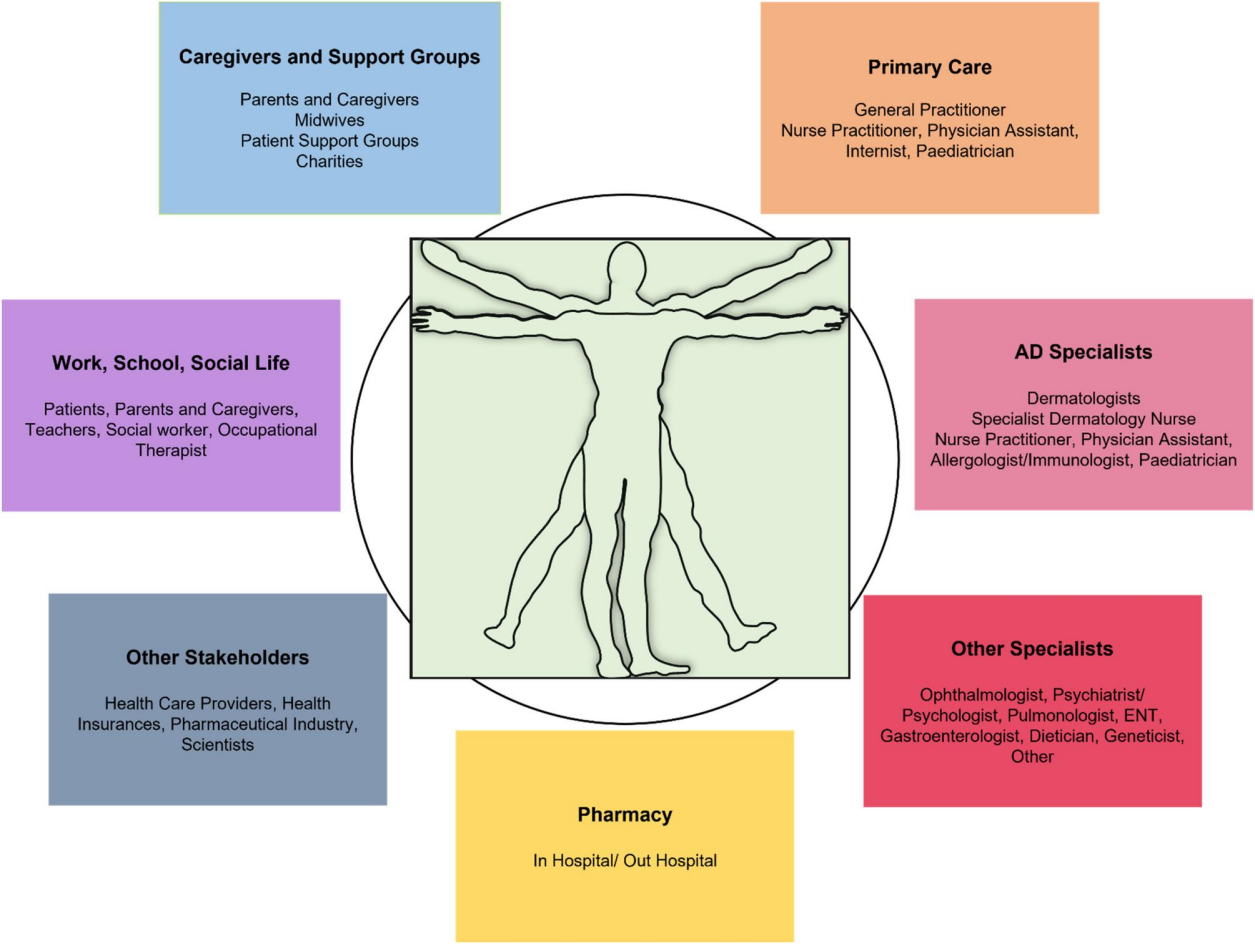
The atopic dermatitis multidisciplinary team.

The document involves all stakeholders: the patient, the pharmacy, the nurses, the doctors, the general practitioner, the paediatrician, the specialist, the tertiary referral centre, the hospitals, academic research institutions, the pharmaceutical industry and patient organisations (Figure 2). Additional stakeholders not involved in the preparation of the document but included as discussion partners are healthcare institutions, healthcare providers and policy makers.

The document also supports the EU efforts on AHA with GA^2^LEN being a partner of the European Innovation Partnership (EIP) initiative on AHA.

ICP Action Plan

(1) Six working groups have developed specific topics identified as particular focus areas (Table 2).
(2) Based on the results of these working groups, the ICPs will then be comprised following a structure with boxes indicating the different levels at which certain knowledge and interventions are required. As an example, regarding diagnostics, the ICPs could provide certain questions that may help the pharmacist to guide the patient as well as further questions and diagnostic procedures to be employed at the GP level. In this context, it will be important to recognise and consider country‐specific variability in professional training and healthcare structure, for example, the different role of GPs and pharmacists or the availability of specialists in different countries.
(3) Guidelines provide evidence but are not always useful in daily practice. In ICPs, the important practical aspects of different guidelines can be combined to improve clinical care. While guidelines should cover the whole spectrum, ICPs summarise the results of these guidelines and focus on the interventions that are mainly used. Thus, it is deemed neither necessary nor appropriate to mention treatment options like intravenous immunoglobulins in AD‐ICPs. Instead, a reference in the box for the specialist may be inserted, indicating that further, rarely‐used treatment options are discussed in the guideline. However, a concluding chapter at the end of the AD‐ICPs will discuss unmet needs and the potential of emerging therapies which are still currently being investigated in clinical trials.

**TABLE 2:** Six working groups developing specific topics considered in the ICPs.

Group 1	**Diagnostic measures**
	Charlotte G Mørtz et al.
	To discuss treatment targets based on clinical scores versus symptoms and markers of quality of life with a focus on the feasibility in a daily practice setting considering geographical variations of healthcare systems
Group 2	**Topical treatment**
	Andreas Wollenberg et al.
	To discuss indications and practicalities as recommended by the guidelines, transitioning points to systemic therapies and the current quality and needs of healthcare provision of topical treatment in AD considering geographical differences
Group 3	**Systemic treatment**
	Thomas Werfel et al.
	To discuss indications for systemic therapies in different patient subgroups and age groups, availability of approved and reimbursed therapies with country‐specific differences as well as the role of off‐label drugs
Group 4	**Comorbidities**
	Wojciech Francuzik et al.
	To discuss atopic and non‐atopic comorbidities and their impact on disease burden and treatment options
Group 5	**Ocular comorbidities**
	Marjolein de Bruin‐Weller et al.
	To discuss the risk of ocular involvement in AD, its assessment and management as well as referral criteria to the ophthalmologist
Group 6	**ICP by digital harmonisation**
	Jean Bousquet
	To discuss digitally‐supported ICPs as part of a patient‐centred approach linking patients, pharmacists and physicians, and an opportunity to lead to a more rapid diagnosis, to guide stepwise treatment in everyday life and to collect patients' feedback

## 5 SUMMARY

The GA^2^LEN ADCARE network has outlined the unmet needs in AD and provided an action plan for devising an AD ICP, with a special focus on involving all stakeholders and on considering the complexity and variability of the disease. With this initiative, we aim to complement existing evidence‐based guidelines while bridging the gaps that often appear in daily clinical practice.

## AUTHOR CONTRIBUTIONS

**Torsten Zuberbier**: conceptualization (lead); data curation (lead); formal analysis (lead); funding acquisition (lead); investigation (lead); methodology (lead); project administration (lead); resources (equal); software (equal); supervision (lead); validation (equal); visualization (equal); writing – original draft (equal); writing – review and editing (equal). **Lisa A. Beck**: methodology (equal); validation (equal); writing – original draft (equal). **Anna Bedbrook**: writing – original draft (equal); writing – review and editing (equal). **Marjolein de Bruin‐Weller**: methodology (equal); validation (equal); writing – original draft (equal). **Jean Bousquet**: methodology (equal); validation (equal); writing – original draft (equal). **Michael Cork**: methodology (equal); validation (equal); writing – original draft (equal). **Nikolaos Douladiris**: formal analysis (equal); methodology (equal); writing – original draft (equal). **Norito Katoh**: methodology (equal); validation (equal); writing – original draft (equal). **Charlotte G. Mortz**: methodology (equal); validation (equal); writing – original draft (equal). **Thomas Werfel**: methodology (equal); validation (equal); writing – original draft (equal). **Francuzik Wojciech**: methodology (equal); validation (equal); writing – original draft (equal). **Andreas Wollenberg**: methodology (equal); validation (equal); writing – original draft (equal); writing – review and editing (equal). **Kristina Siemens**: conceptualization (lead); methodology (equal); visualization (equal); writing – original draft (equal); writing – review and editing (equal). **Katarina Stevanovic**: conceptualization (equal); data curation (equal); methodology (equal); project administration (lead); visualization (equal); writing – original draft (equal); writing – review and editing (lead). **Margitta Worm**: methodology (equal); validation (equal); writing – original draft (equal).

## CONFLICT OF INTEREST STATEMENT

Torsten Zuberbier has received institutional funding for research and/or honoria for lectures and/or consulting from AstraZeneca, AbbVie, ALK, Almirall, Astellas, Bayer Health Care, Bencard, Berlin Chemie, FAES, HAL, Henkel, Kryolan, Leti, L'Oreal, Meda, Menarini, Merck, MSD, Novartis, Pfizer, Sanofi, Stallergenes, Takeda, Teva and UCB; in addition, he is a member of ARIA/WHO, DGAKI, ECARF, GA^2^LEN and WAO. Lisa A. Beck has no conflict of interest to declare. Anna Bedbrook has no conflict of interest to declare. Marjolein de Bruin‐Weller has been a consultant, advisory board member and/or speaker for AbbVie, Almirall, Eli Lilly, Galderma, Janssen, Leo Pharma, Pfizer, Regeneron, Sanofi‐Genzyme and UCB. Jean Bousquet reports lecture honoraria from Cipla, Menarini, Mylan, Novartis, Purina, Sanofi‐Aventis, Teva, Uriach; outside the submitted work, and is a shareholder of KYomed Innov and MASK‐air‐SAS. Michael Cork is an investigator and consultant for Regeneron, Sanofi Genzyme, Pfizer, Leo, Galapagos, Novartis, Boots, L'Oreal, Dermavant, Menlo, Reckitt Benckiser, Oxagen, Johnson & Johnson, Hyphens, Astellas, AbbVie, Galderma and Procter & Gamble. Nikolaos Douladiris has no conflict of interest to declare. Norito Katoh reports grants and personal fees from Mitsubishi Tanabe Pharm, Taiho Pharmaceutical, Torii Pharmaceutical, Maruho, Kyowa Kirin, Ely‐Lilly Japan, Sanofi, Jansen Pharma, Eisai, A2 Healthcare, Leo Pharma, Boehringer Ingelheim Japan, Abbvie, Celgene Japan. Charlotte G. Mortz has no conflict of interest to declare. Thomas Werfel reports grants and personal fees from Sanofi Regeneron, Leo Pharma, Ely Lilly, Abbievie, Pfizer, Almirall, Galderma, and Novartis. Francuzik Wojciech has no conflict of interest to declare. Andreas Wollenberg reports personal fees from AbbVie, Chugai, Galderma, LEO Pharma, Lilly, MedImmune, Novartis, Pfizer, Regeneron and Sanofi‐Aventis; and grants from LEO Pharma outside the submitted work. Kristina Siemens reports personal fees for medical writing from the GA^2^LEN ADCARE Network. Katarina Stevanovic has no conflict of interest to report. Margitta Worm reports grants and personal fees from Stallergens, HAL Allergie, Bencard Allergie, Allergopharma, ALK‐Abello, Mylan Germany, Actelion Pharmaceuticals Deutschland, Biotest, AbbVie Deutschland, Lilly Deutschland Aimmune, DBV Technologies, Regeneron Pharmaceuticals, Sanofi Aventis, Leo Pharma, Novartis and Viatris, outside the submitted work.

## Data Availability

Data sharing not applicable to this article as no datasets were generated or analysed during the current study.
